# How is and how should healthcare for people with multiple sclerosis in Germany be designed?–The rationale and protocol for the mixed-methods study Multiple Sclerosis–Patient-Oriented Care in Lower Saxony (MS-PoV)

**DOI:** 10.1371/journal.pone.0259855

**Published:** 2021-11-11

**Authors:** Kathrin Krüger, Lara Marleen Fricke, Elise-Marie Dilger, Annett Thiele, Kristina Schaubert, Dyon Hoekstra, Fedor Heidenreich, Anna Levke Brütt, Jona T. Stahmeyer, Alexander Stahmann, Anna-Lena Röper, Klaus-Peter Kubiak, Melissa Hemmerling, Anja Grau, Kerstin Eichstädt, Sabine Behrens, Christian Krauth

**Affiliations:** 1 Institute for Epidemiology, Social Medicine, and Health Systems Research, Hannover Medical School (MHH), Hannover, Germany; 2 Center for Health Economics Research (CHERH), Hannover, Germany; 3 Department für Versorgungsforschung, Carl von Ossietzky Universität Oldenburg, Oldenburg, Germany; 4 Institut für Sonder- und Rehabilitationspädagogik, Carl von Ossietzky Universität Oldenburg, Oldenburg, Germany; 5 Klinik für Neurologie und klinische Neurophysiologie, DIAKOVERE Henriettenstift, Hannover, Germany; 6 Deutsche Multiple Sklerose Gesellschaft (DMSG) Landesverband Niedersachsen e.V., Hannover, Germany; 7 Health Services Research Unit, AOK Niedersachsen, Hannover, Germany; 8 MS Forschungs- und Projektentwicklungs-gGmbH, Hannover, Germany; Public Library of Science, UNITED KINGDOM

## Abstract

**Background:**

Multiple sclerosis (MS) is the most common autoimmune inflammatory disease of the central nervous system in Europe, often causing severe physical, cognitive and emotional impairments. Currently, it is unclear whether the healthcare provisions of people with MS (PwMS) are in line with the recommendations for treatment based on guidelines or patients’ needs. The main objectives of the study are as follows: (a) to investigate how well PwMS are treated; and (b) to develop a needs-oriented, patient-centred care model.

**Methods:**

This mixed-methods study focuses on adult PwMS living in Lower Saxony, a federal state in Germany. The qualitative study comprises focus groups with PwMS, physicians and people involved in the healthcare process as well as a future workshop. The quantitative study comprises a cross-sectional online survey and addresses the patient-relevant outcomes and needs, as previously determined by literature searches and focus groups. It will be administered to all PwMS who are insured by the statutory health insurance company involved in the project (n~7,000). The survey data will be linked to the longitudinal secondary data from the statutory health insurance company and data from the German MS registry where available. The linked and single data sources will be statistically analysed.

**Discussion:**

By comprehensively comparing the current healthcare provisions with the needs and requirements of PwMS, the strengths and weaknesses of the overall healthcare process and provision of assistive devices can be identified. The barriers and facilitators of the health service providers and their impact on daily life will be explored (qualitative analyses). Reliable recommendations for improvements will be given based on a study population drawn from the largest statutory health insurance company in Lower Saxony (quantitative analyses). However, the inherent advantages and limitations of the qualitative and quantitative research approaches need to be considered.

**Trial registration:**

The study is registered at German Clinical Trials Register DRKS00021741.

## Introduction

Multiple sclerosis (MS) is the most common autoimmune inflammatory disease of the central nervous system in Europe. Depending on the region, population and methodology, studies report up to 253/100,000 people in crude prevalence and 18/100,000 in crude incidence [1]. As there are more than 220,000 people with MS (PwMS) in Germany, the annual incidence is approximately 18/100,000 in statutory insured persons [2]. MS leads to severe physical, cognitive and emotional impairments [3]. The disease often manifests between the ages of 20 and 40 years [4]. In over 80% of PwMS, the disease begins with a relapsing-remitting course. Eventually, without appropriate medical care, continuous (secondary) progression of the neurological symptoms occurs in at least 50% of cases. Approximately 10% of all PwMS experience a gradual deterioration from the onset of the disease (primary progression) [5, 6].

Guidelines can be used to assess the current quality of healthcare, as these recommendations ensure adequate healthcare in defined settings. Therefore, the level of adherence to guidelines by healthcare providers may serve as an external criterion for the appropriateness of the current healthcare provisions regarding PwMS [7]. The guideline by the German Society of Neurology describes the best possible evidence-based healthcare, such as in the fields of diagnostics, therapy and reproductive medicine, or subgroups based on age [8, 9].

Although the provision of assistive devices is important, as approximately one in three PwMS report having received at least one assistive device during the last 12 months [10], it is barely covered in the current guideline. Assistive devices that are used by PwMS are predominantly mobility-related, such as walking aids or wheelchairs [10, 11]. Studies show that assistive devices increase functional capacity and patient-oriented outcomes, such as quality of life and participation [11, 12]. The correct use of the appropriate assistive devices benefits PwMS [13, 14]. However, many PwMS either do not use the prescribed assistive devices or only use them sporadically [14]. By conducting a detailed multi-professional assessment, provisions according to the individual’s needs can be assessed, which would promote the use of assistive devices [15].

In general, costs related to MS include indirect (e.g. production losses) and direct costs, more precisely healthcare costs, especially for disease-modifying therapies, and non-healthcare costs (e.g. community services and informal care). The annual mean costs, as calculated by a large European study, ranged from €22,800 to €57,500 for people with mild and severe MS, respectively [4]. In Germany, the annual direct costs of illness (healthcare, services and informal care costs) are estimated to be €20,024 for those with mild MS and €41,149 for those with severe MS. Although the costs generally increase according to the disease stage, the costs for disease-modifying therapies, which dominate in early (relapsing) disease stages, decrease (mild: €15,819; severe: €4,981). The annual indirect costs due to short- and long-term absenteeism from work, disability and early retirement vary between €8,190 (mild) and €21,586 (severe) [10]. This demonstrates the influence of MS, especially on health insurance funds. Healthcare insurance is mandatory in Germany, and healthcare costs (e.g. pregnancy, maternity, prevention and treatment of diseases) are comprehensively covered by (statutory) health insurance funds [16]. Most (approximately 88.2%) of the German population is statutorily insured (n~69,753,000) [17].

This protocol describes the study “Multiple Sclerosis–Patient-Oriented Care in Lower Saxony (MS-PoV)” (German: Multiple Sklerose–Patientenorientierte Versorgung in Niedersachsen [MS-PoV]) in detail. The objective of the study is to evaluate the current healthcare provisions for PwMS in Lower Saxony, thereby comparing it with the guideline’s recommendations and the self-reported subjective needs of PwMS.

## Materials and methods

### Study aim and setting

The overall aims of the project are as follows: (a) to investigate how well PwMS are cared for from different perspectives (e.g. patients’ and medical perspectives based on the guideline’s recommendations); and (b) to develop a needs-based, patient-centred care model. The study is funded from 1 April 2020 to 31 March 2023 and captures the following two thematic focuses: a general analysis of the healthcare for PwMS (focus 1); and a detailed analysis of the healthcare regarding assistive devices (focus 2). In addition to the study’s primary objective–quality of care from the patient’s perspective–the research questions below comprehensively address these focuses.

### Focus 1: Research questions addressing general healthcare for PwMS

1.1 What do PwMS in Lower Saxony objectively need (following guideline-adherent care, e.g. the demand of subgroups and needs of PwMS)?
1.2 What healthcare structures and processes currently exist in Lower Saxony (e.g. number and regional distribution of neurologists, waiting period)?
1.3 What healthcare services are claimed by PwMS (for both outpatient care, such as general practitioners and specialists, pharmaceuticals, therapeutic treatment and assistive devices, and for inpatient care), and are there any differences between subgroups, such as regional differences?
1.4 How rate PwMS patient-relevant outcomes (e.g. health-related quality of life [HRQoL], general state of health, satisfaction with healthcare)?
1.5 Do the existing support structures meet the objective needs of PwMS?
1.6 Is clinical practice consistent with the guideline’s recommendations?
1.7 How high are the current expenses (in total and per service area) in Lower Saxony, and what is the cost of needs-based care?

### Focus 2: Research questions addressing healthcare regarding assistive devices

2.1 Which assistive devices are prescribed and/or obtained during the course of the disease (at which time points and for which impairments)? How is the provision process of mobility assistive devices perceived and how well do mobility assistive devices match the needs of PwMS?
2.2 How do PwMS and professionals describe the current provision process of assistive devices? How satisfied are PwMS with the provision of assistive devices? What needs do PwMS express regarding the provision of assistive devices?
2.3 How is the demand for the provision of assistive devices determined? What (international) concepts can be identified?
2.4 What does a demand-based supply of assistive devices look like?

The immediate results of this project are expected to be reliable statements regarding patients’ needs; care structures and processes; utilisation of services; and quality of care. The subjective needs (and wishes) of PwMS will be compared with the current structures and processes to determine the gaps in the healthcare system. As Lower Saxony, which is one of the 16 federal states in Germany, is characterised by a mixture of rural, urban and metropolitan regions, the analysis of the project focuses on the regional differences and compares different subgroups (e.g. type and severity of MS or sex).

### Study design

**Fig 1:**
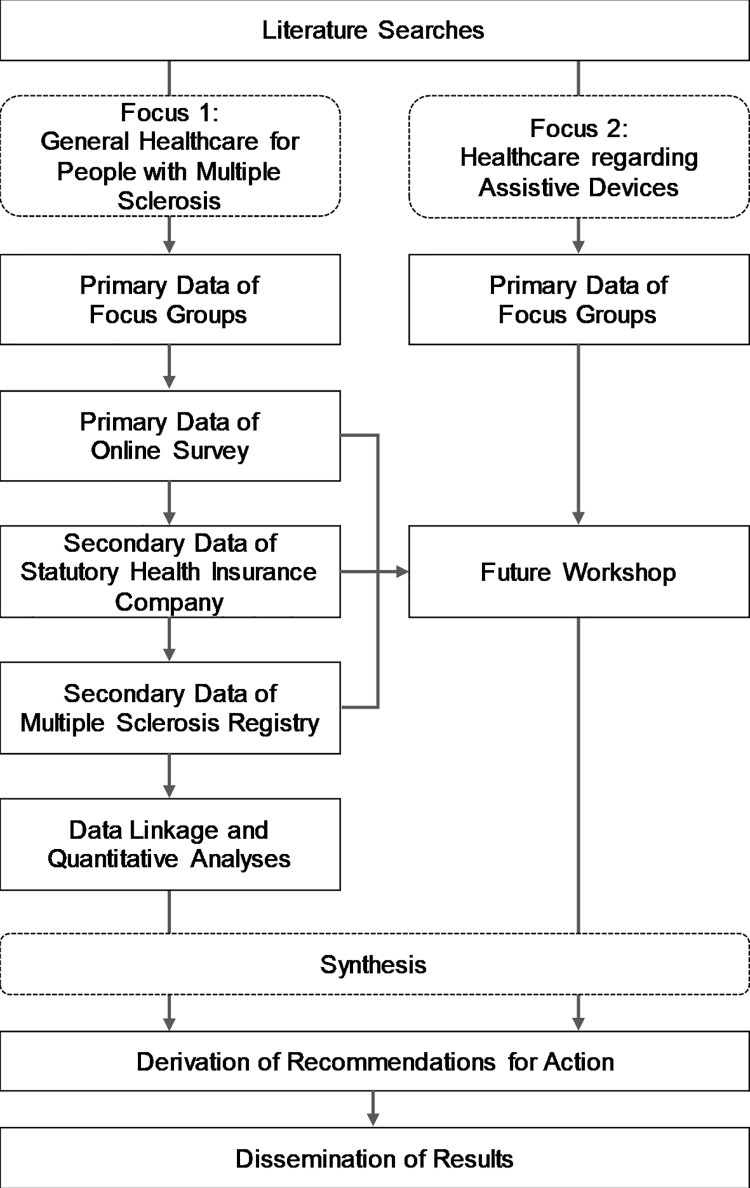
Interaction of data sources and thematic focuses.

The mixed-methods design uses both qualitative and quantitative research approaches (Table 1). Initially, literature searches were conducted as a basis for the subsequent work packages to address various key aspects of the project. The general healthcare for PwMS (focus 1) is analysed using focus groups comprising PwMS and physicians who are involved in the healthcare for PwMS; a cross-sectional online survey; and secondary data analyses using data from the largest statutory insurance company in Lower Saxony (AOK Lower Saxony) and the German MS registry. The provision of assistive devices (focus 2) is investigated using focus groups comprising PwMS, physicians and other professionals who are involved in the process of providing assistive devices to PwMS (e.g. occupational therapists, physiotherapists, employees of medical supply stores), along with quantitative data as described in focus 1. Using the participatory method of a future workshop [18], the assistive device provision process is critically discussed based on the findings of previously conducted focus groups and secondary data analyses. This will provide recommendations for the short, medium and long term (Fig 1).

**Table 1:** Data sources.

Objective	Research questions*	Specifying the data source	Target group and planned number of participants (n)	Data source
***Literature Search***				
Analyses of the general healthcare for PwMS (focus 1) and healthcare regarding assistive devices (focus 2)	1.1, 2.3	-	-	-
***Qualitative data***				
Analysis of the general healthcare for PwMS (focus 1)	1.1, 1.4	Focus groups	PwMS (24)^a^	Primary data
			Physicians (8)^b^	
Analysis of the healthcare regarding assistive devices (focus 2)	2.2	Focus groups	PwMS (32)^c^	Primary data
			Physicians and other professionals (32)^d^	
Analysis of the healthcare regarding assistive devices (focus 2)	2.4	Future workshop	PwMS (15)	Primary data
			Physicians and other professionals (10)	
***Quantitative data***				
Analyses of the general healthcare for PwMS (focus 1) and healthcare regarding assistive devices (focus 2)	1.1, 1.4, 2.1	Online survey	PwMS (7,000, response 25%: 1,750)	Primary data
	1.2, 1.3, 2.1	Secondary care data of a statutory health insurance company	PwMS (1,750, depending on response to online survey)	Secondary data
	1.2, 1.3, 2.1	MS registry data	PwMS (800)	Secondary data
	*1*.*1–1*.*7*, *2*.*1*	*Data linkage*	*PwMS taking part in online survey*	*Primary and secondary data*
Analyses of the general healthcare for PwMS (focus 1) and healthcare regarding assistive devices (focus 2)–analyses of single data sources	*1*.*1–1*.*7*, *2*.*1*	Secondary care data of a statutory health insurance company	PwMS (7,000)	Secondary data

*As listed in the study aims and setting above.

^a^3 focus groups with 8 participants each.

^b^1 focus group with 8 participants^.^

^c^4 focus groups with 8 participants each.

^d^4 focus groups with 8 participants each.

MS: Multiple Sclerosis; PwMS: People with Multiple Sclerosis.

#### Literature searches

Initially, a literature search was conducted to systematically collect the international and national status quo of healthcare for PwMS. For this purpose, the relevant databases (e.g. Pubmed/Medline, Web of Science, CINAHL database, Guidelines International Network) were searched. The literature search focused on the following aspects: (1) (inter)national guidelines; (2) subjective needs of PwMS; (3) assistive devices and social support; (4) healthcare provisions, structures and processes; (5) quality of healthcare; (6) preferences of patients regarding healthcare; and (7) health economic aspects.

#### Focus 1: Data used to analyse general healthcare for PwMS–primary data of the focus groups

Focus groups were planned according to the literature to capture the relevant aspects of the healthcare provision and subsequent needs of PwMS [19]. In November and December 2020, three focus groups with PwMS and one with physicians were conducted using an online conference tool. Semi-structured interview guides were used by two trained moderators. The interview guides were derived from the results of the literature search. Furthermore, the interview guide that was used in the focus groups with PwMS was pretested with PwMS and revised as necessary. During the focus groups, technical support was installed and was available to the participants over the phone if any technical issues occurred.

#### Focus 1: Data used to analyse general healthcare for PwMS–primary data of the online survey

The entire study population is surveyed in a quantitatively comprehensive manner in autumn 2021. The questionnaire addresses socio-demographic and socio-economic factors; questions regarding the healthcare provisions and associated potentials and barriers; and the needs, wishes and satisfaction level of PwMS. Furthermore, it investigates the general circumstances of daily life of PwMS. For this purpose, the subjectively perceived state of health; HRQoL; comorbidities, such as depression or fatigue; and impairments in everyday life are surveyed.

Standardised instruments were chosen based on the results of the literature searches (Table 2) [20–44]. The assessment of the quality of care from the patient’s perspective, which is the study’s primary objective, is defined using the Patient Assessment of Chronic Illness Care (PACIC)–Short Form [23, 24]. This standardised instrument includes 11 items regarding the patients’ experience of the treatment of a chronic disease. The 12th item addresses their overall satisfaction regarding the healthcare of a chronic disease. The differences between rural, urban and metropolitan regions, sex, severity of MS and the course of MS will be examined. When no eligible standardised instrument was available, additional questions were developed. These questions were derived from the findings of focus groups and by modifying further instruments identified in literature searches [4, 10, 45–55].

**Table 2:** Instruments used in the online survey.

Concept	Instrument (abbreviation)
Health related Quality of Life	EQ-5D-5L [20, 42, 43]
Quality of care from the patient’s perspective	Patient Assessment of Chronic Illness Care (PACIC)–Short Form [23, 24]
Degree of control for individuals when decisions are being made regarding medical treatment	Computerized Control Preference Scale (eCPS) [21]
Impact of MS on day-to-day life	Multiple Sclerosis Impact Scale (MSIS-29) [22]
Disability and walking ability in Multiple Sclerosis	Patient Determined Disease Steps (PDDS) [25–27]
Social support	Brief Social Support Scale (BS6) [28]
Social participation	Short Scale Measuring Perceived Social Participation (KsT-5) [32]
	World Health Organization Disability Assessment Schedule 2.0 (WHODAS 2.0) [33, 44]
Self-efficacy	Generalized Self Efficacy scale (GSE) [29, 30]
Self-efficacy regarding use of the assistive devices	self-efficacy regarding assistive device use scale developed by Roelands et al. [41]
Reasons for non-use of an assistive device	Assistive Technology Device Predisposition Assessment (ATD PA) [38–40]
Evaluation of the assistive device delivery process	“Kwaliteit van Zorg” (KWAZO) [36, 37]
Satisfaction with the assistive device	Device subscale from the Quebec User Evaluation of Satisfaction with Assistive Technology (QUEST 2.0-G) [34, 35]

To check the comprehensibility and redundancy of the questionnaire, comprehensive cognitive pre-tests according to the concurrent think-aloud method were conducted [56]. The resulting revision of the questionnaire was likewise pretested. As an incentive for taking part in the survey, the DMSG Lower Saxony organises a day trip to a local zoo for 25 randomly chosen families of participating PwMS.

#### Focus 1: Data used to analyse general healthcare for PwMS–secondary data from a statutory health insurance company

Secondary data from a statutory health insurance company is collected for billing purposes and includes information on socio-demography, comorbidities, longitudinal utilisation of services and cost data for various aspects of healthcare for PwMS (e.g. outpatient and inpatient diagnoses, prescribed pharmaceuticals or sick leave). With this data source, the differences in healthcare utilisation between various patient groups can be identified.

#### Focus 1: Data used to analyse general healthcare for PwMS–secondary data from the MS registry

Data included in the MS registry is transferred to the MS registry by MS specialised centres in the context of MS therapy. This data source comprises clinical information regarding the medical history, socio-demographics and initial symptoms, in addition to the follow-up data and information on symptoms, therapy, care, medication and relapses. The German MS Registry is described in more detail by Ohle et al. [57].

#### Data linkage

In addition to analyses within single data sources, the secondary data from the statutory insurance company will be linked to the data from the online survey and secondary data from the MS registry. However, as only MS specialised centres report to the MS registry, and enrolment in the MS registry is not mandatory, not all PwMS are listed in this registry.

#### Focus 2: Data used to analyse healthcare regarding assistive devices–primary data from the focus groups

Eight focus groups (four focus groups with PwMS and four with physicians and other professionals) were created to explore the experiences and needs regarding the provision of assistive devices. The discussion guide was pretested and subsequently adapted. The focus groups were conducted as described earlier to analyse the general healthcare for PwMS (focus 1). Four focus groups with PwMS were conducted in May 2021. To complement these findings, four focus groups with professionals involved in the provision of assistive devices for PwMS, namely neurologists, occupational therapists, physiotherapists, general practitioners, (MS) nurses and employees of medical supply stores, were conducted in September 2021. Participants could express their target group-specific knowledge of the assistive device provision process and name and explain the barriers and factors regarding successful assistive device provision and use in the presence of MS.

#### Focus 2: Data used to analyse healthcare regarding assistive devices–quantitative data sources

To evaluate the provision process of assistive devices, relevant quantitative data sources (primary data from the online survey, secondary data from the statutory health insurance company, secondary data from the MS registry) will be analysed.

#### Focus 2: Data used to analyse healthcare regarding assistive devices–future workshop

Healthcare regarding assistive devices will be critically discussed based on the findings from the previously conducted literature search, focus groups and quantitative data analyses in 2022. The findings of the future workshop will guide the development of an assistive device provision model that aims to optimally adapt to the specific needs of the target group. The future workshop is a face-to-face method with a group size of a maximum of 25 participants [18]. The future workshop consists of the following three phases: (1) complaints and criticism; (2) fantasy and utopia; and (3) realisation and practice.

#### Qualitative research approaches–study population

PwMS who were 18 years of age or older, members of the DMSG Lower Saxony and had a confirmed diagnosis according to the McDonald criteria [58] were recruited for the focus groups and pre-tests for the online survey. In the latter case, only PwMS not insured by AOK Lower Saxony were included. Furthermore, due to the qualitative research approach, only study participants who could understand and speak German sufficiently were included. The focus group participants were selected based on purposeful sampling. Focus groups with PwMS should include persons with different characteristics (e.g. age, sex, region of residence, degree of impairment and duration of illness). Additional focus groups with physicians and those involved in care should vary in age, sex, region and profession. PwMS recruitment was carried out by DMSG Lower Saxony, and the recruitment of physicians was supported by the Association of Statutory Health Insurance Physicians Lower Saxony (German: Kassenärztliche Vereinigung Niedersachsen). Other professionals in the care process (e.g. occupational therapists, physiotherapists, employees of medical supply stores) were recruited through further professional associations (contact establishment via DMSG Lower Saxony). Due to the qualitative research approach, the sample cannot be representative. However, it is important to include participants with different roles in the care process to capture a broad insight in the current healthcare for PwMS.

#### Qualitative research approaches–data analysis

The focus groups were digitally recorded, transcribed and were and will be subsequently evaluated using a content analysis [59]. A qualitative analysis of the focus groups was and will be conducted using the software MAXQDA. A protocol of the recommendations and ideas resulting from the future workshop will be created.

#### Quantitative research approaches–study population

All statutory insured persons of the above mentioned statutory insurance company (AOK Lower Saxony) that were diagnosed with relapsing or progressing MS (ICD.-10 G35.-) in 2019 or 2020 are included in the quantitative study. To define the study population, the following further refinement of the criteria for a MS diagnosis applies: (a) at least two diagnoses in two different quarters in the outpatient setting (M2Q-criterion); (b) at least one outpatient diagnosis with additional course-modifying MS therapy; or (c) at least one outpatient diagnosis with at least one diagnosis in the inpatient setting (as the principal or secondary diagnosis). Diagnosed PwMS are eligible if they are at least 18 years of age and live in the federal state of Lower Saxony. All eligible PwMS (n~7,000) will be included in the secondary data analysis of the statutory insurance data. Amongst those, all are invited to take part in the online survey by written letters. Additionally, for those taking part in the online survey (anticipated response: 25%: n~1,750), the survey data will be linked with the secondary data from the statutory insurance company and MS registry.

#### Quantitative research approaches–sample size calculation for the online survey

The sample size calculation is based on the study’s primary endpoint, as defined by the PACIC–Short Form [23, 24]. Since there is limited information on MS healthcare and as this study is limited to the federal state of Lower Saxony, there is insufficient information on effect sizes in the literature. Therefore, the sample size calculation aims at being able to detect even small effects [60]. In order to avoid an alpha-error accumulation by multiple testing (four tests), the following Bonferroni correction [61] of the significance level is applied in the analysis: alpha_adj_ = alpha/N_tests_ = 0.05/4 = 0.0125. For a test design with a power of 90% (1—beta = 0.9) and a significance level of 1.25% (alpha_adj_ = 0.0125), a case number of 1,432 subjects is required for the significant detection of a small difference (effect strength d = 0.2) [62]. Considering a dropout of 75%, this results in a sample size of 5,728 (required case number of 1,432 subjects + expected dropout of 4,296 subjects = 5,728 subjects). The sample size of approximately 7,000, which compromises the entire population of insured PwMS by AOK Lower Saxony who fulfil the inclusion criteria, will be sufficient for evaluations according to various strata (e.g. age, sex, place of residence).

#### Quantitative research approaches–data analysis

The resulting health outcomes that will be evaluated by descriptive analyses are for example patient-relevant outcomes, such as HRQoL, the number of relapses per year and satisfaction with MS healthcare. Correlational analyses will be used to examine associations with parameters, such as healthcare service utilisation and costs (and others). Regional differences in healthcare service utilisation and the resulting health outcomes will be analysed using confirmatory statistical methods (such as inferential statistical tests) and multivariate regression models.

Based on the guideline recommendations [8, 9] and the subjective needs of PwMS (drawn from the online survey), model estimates will be calculated to determine the need for healthcare provisions and the associated costs of needs-based healthcare in Lower Saxony. The total number of PwMS; the distribution of forms and severity; and the average number of relapses will be included in the model.

Finally, whether the supply of care in Lower Saxony meets the needs of PwMS will be analysed by comparing the existing resources and requirements. Analyses will be carried out using the software programs SAS, R and SPSS and Microsoft Excel.

#### Ethical considerations and data management

This study was approved by the Ethics Committee of the Hannover Medical School on 24 June 2020 (reference number 9173_BO_K_2020) and by the University of Oldenburg on 06 August 2020 (reference number 2020–108). Whenever necessary, approval of changes of the initial study protocol was and will be obtained from the ethics committees of Hannover Medical School and/or University of Oldenburg. The study is and will be conducted in accordance with the principles of the Declaration of Helsinki. The principles of ‘Good Clinical Practice’ and all relevant legal, ethical and data protection principles will be adhered to. All persons actively participating in the study will be fully and comprehensibly informed about the project’s purpose and procedure and the handling of the collected data. Whenever necessary, the research team obtains informed consent for the qualitative (written consent) and quantitative (online consent) research approaches regarding the legal, ethical and data protection principles. Participation in the study is voluntary and can be withdrawn at any time. Data already collected will then be deleted. Non-participation has no consequences. Data will be deleted ten years after the end of the project.

Details regarding the qualitative research approaches: The participants of the focus groups and the future workshop will be informed that their participation is voluntary and that they have a right to refuse or withdraw from the study without any consequences. The audio-recorded data will be transcribed and analysed without personal information and deleted after the transcription is complete. Access to data is limited to selected members of the project team.

Details regarding the online survey: The statutory health insurance company invites insured persons who fulfil the inclusion criteria. The MS registry, which is another project partner, hosts the online survey and informs the statutory health insurance company using pseudonyms to ensure the confidentiality of the collected data and allow non-responder analyses. The statutory health insurance company will not receive any information from the completed questionnaire, and the MS registry will not obtain any of the participants’ personal information, apart from the answers provided in the online survey. The Hannover Medical School receives pseudonymised data from the statutory health insurance company, the online survey and the MS registry for linkage and evaluation. Linkage is possible using pseudonyms and a matching table that were previously generated by an independent trust agency. However, project partners that evaluate the linked data will not receive any information that could result in the participants being identified. In accordance with § 75 of the German Social Code, Book X (SGB X), the transfer of the claims data for the research purpose will be legitimated by the competent supervisory authority (Ministry for Social Affairs of Lower Saxony; German: Niedersächsisches Ministerium für Soziales, Gesundheit und Gleichstellung). Access to data is limited to selected members of the project team.

#### Derivation of recommendations for action

Considering the results of the literature searches, focus groups, online survey, secondary data and future workshop, recommendations for action to improve the healthcare for PwMS will be developed within a workshop, which will involve all project partners and experts who are advising the entire project.

The project aims to generate reliable statements regarding the needs for care; current care structures and processes; utilisation of healthcare services; and resulting health outcomes. The results of the study will contribute to the identification of the differences between rural, urban and metropolitan regions and deficits in care. The analyses, alongside the statutory health insurance data and information from those involved in treatment structures, allow the optimisation of healthcare for PwMS, including pharmaceutical therapy, other therapeutic measures (e.g. physiotherapy, speech and occupational therapy) and assistive devices. In rural, urban and metropolitan regions, the infrastructure can be optimised in a patient-oriented manner for all areas of life affected by MS. The results and models can form the basis to pilot and evaluate a new form of healthcare.

## Discussion

To the best of our knowledge, this is the first study to consider and link various data sources (linkage of quantitative data: secondary data of statutory insurance and MS registry data with primary data from the online survey; and qualitative data sources: focus groups and future workshop) to determine the current healthcare provision of PwMS in Germany and how it is perceived by those affected based on a large patient population. The strength of the project is derived from the analyses of different data sources and perspectives from various research approaches. The patients’ perspective is complemented by the perspective of professionals in the context of healthcare for PwMS and the secondary data from the statutory health insurance company and MS registry. This enables precise recommendations regarding the optimisation of healthcare for PwMS, including pharmaceutical therapy, other non-physician therapies (physiotherapy, speech and occupational therapy) and assistive devices. The results and model suggestions can form the basis to pilot and evaluate a new form of healthcare. As Lower Saxony contains a mixture of rural, urban and metropolitan regions, we expect that our results can be used as a template for similar regions in Germany to optimise the healthcare for PwMS outside of the studied region.

The main methodological challenges in this project relate to the representativeness of the of the recruited study population. The qualitative approaches do not aim to generate representative findings; instead, they aim to identify thematic areas for further investigation. Therefore, the goal of the online survey is to obtain reliable statements from the patients’ perspective regarding healthcare for PwMS in Lower Saxony. A possible selection bias must be investigated. Focusing on this aspect, one study published in 2013 [63] showed no significant differences in the age and sex distribution between those insured by AOK Lower Saxony and the general population of Germany. However, on average, although those insured by AOK Lower Saxony differ in terms of occupation from the general population, in the group of PwMS, higher occupational groups should be sufficiently represented to enable accurate analyses. Additionally, a non-responder analysis will be conducted and, if necessary, further computational adjustments will be made to the data set. Therefore, the basic information (e.g. age and sex) of those taking part and not taking part in the survey will be compared. Moreover, whether there is a significant difference between those groups will be checked using a t-test or Chi^2^-test.

To proactively minimise a possible participation bias, the study is promoted using various platforms of the DMSG Lower Saxony and AOK Lower Saxony. The invitation letters for the online survey are developed in close coordination with the DMSG Lower Saxony, and postal reminders are sent out after two and four weeks. Furthermore, a telephone contact will be offered.

Study results will be disseminated via national and international conference presentations and publications in peer-reviewed journals. Project partners will additionally publish results of the project in non-scientific language on their homepages and member journals.

MS is a lifelong disease that manifests at a young age in various clinical appearances [4]. Therefore, personalised care according to the individual needs of PwMS is essential to positively influence the progression of MS and ensure a lasting participation in PwMS’s social lives and work. As the progression of the disease can be positively and negatively influenced by factors that may interact, the use of preventive measures is indicated. The identification of barriers in the healthcare sector based on the knowledge gained in this study allows conclusions to be drawn regarding the optimisation of structures and processes in the overall treatment for MS.
